# A Simple and Specific Noncompetitive ELISA Method for HT-2 Toxin Detection

**DOI:** 10.3390/toxins9040145

**Published:** 2017-04-20

**Authors:** Henri O. Arola, Antti Tullila, Alexis V. Nathanail, Tarja K. Nevanen

**Affiliations:** 1 VTT Technical Research Centre of Finland, Tietotie 2 FI-02150 Espoo, Finland; antti.tullila@vtt.fi (A.T.); tarja.nevanen@vtt.fi (T.K.N.); 2 Finnish Food Safety Authority (Evira), Chemistry and Toxicology Unit, Research and Laboratory Department, Mustialankatu 3, FI-00790 Helsinki, Finland; alexis.nathanail@helsinki.fi

**Keywords:** HT-2 toxin, recombinant antibodies, ELISA, noncompetitive, Fab, scFv, alkaline phosphatase, fusion protein, cereal grains

## Abstract

We developed an HT-2 toxin-specific simple ELISA format with a positive read-out. The assay is based on an anti-immune complex (IC) scFv antibody fragment, which is genetically fused with alkaline phosphatase (AP). The anti-IC antibody specifically recognizes the IC between a primary anti-HT-2 toxin Fab fragment and an HT-2 toxin molecule. In the IC ELISA format, the sample is added together with the scFv-AP antibody to the ELISA plate coated with the primary antibody. After 15 min of incubation and a washing step, the ELISA response is read. A competitive ELISA including only the primary antibody recognizes both HT-2 and T-2 toxins. The anti-IC antibody makes the assay specific for HT-2 toxin, and the IC ELISA is over 10 times more sensitive compared to the competitive assay. Three different naturally contaminated matrices: wheat, barley and oats, were used to evaluate the assay performance with real samples. The corresponding limits of detection were 0.3 ng/mL (13 µg/kg), 0.1 ng/mL (4 µg/kg) and 0.3 ng/mL (16 µg/kg), respectively. The IC ELISA can be used for screening HT-2 toxin specifically and in relevant concentration ranges from all three tested grain matrices.

## 1. Introduction

HT-2 toxin (HT-2) and T-2 toxin (T-2) are the most toxic trichothecene mycotoxins [1,2,3]. They occur in grains such as barley, wheat and oats, and are produced by *Fusarium* species [4]. European Union recommendations for the sum of HT-2 and T-2 in unprocessed barley, oats and wheat have been set to 200, 1000 and 100 µg/kg, respectively [5]. Most reports of HT-2 and T-2 occurrence have come from northern Europe, France and the UK, and therefore the problem related to these type A trichothecene mycotoxins has previously been associated with a cool climate [4]. Recently, Morcia et al. [6] reported the first occurrence data of HT-2 and T-2 in Italian malting barley (2011–2014), indicating that type A trichothecene levels can be even higher in southern Europe than in the north, and that the levels of these toxins in barley have been increasing. In 2014, almost 20% of the samples studied exceeded the EU recommendations (200 µg/kg) for the sum of HT-2 and T-2 in barley [6].

In addition to well-established analytical methods using liquid chromatographic (LC) or gas chromatographic (GC) separation combined with mass spectrometry (MS) for mycotoxin determination [7], easier, more rapid and high-throughput screening methods are also needed. The current rapid methods for HT-2 and T-2 are competitive immunodetection methods that include fluorescence polarization [8], magnetic bead-based assays [9], surface plasmon resonance (SPR) [10], lateral flow and enzyme-linked immunosorbent assays (ELISA) [11]. 

ELISA methods are the most used rapid analytical tools in food safety analysis, and they are already commercially available for several mycotoxins [12]. Currently, the detection of mycotoxins with competitive immunoassays is performed using primary or secondary antibodies labelled with, e.g., an enzyme or a fluorescent dye. Horseradish peroxidase (HRP) and alkaline phosphatase (AP) are the most widely used enzyme labels in ELISA. Chemical conjugation of enzymes to proteins results in random location and number of labels, leading to a heterogeneous detection reagent [13]. By using molecular cloning techniques, it is possible to construct homogeneous fusions in which an enzyme is attached to an antibody fragment opposite to the binding site, without disturbing the antibody’s binding properties [14]. Antibody-enzyme fusions also enable single step detection without the need for a secondary antibody [15]. *E. coli* AP is active as a dimer [16] and its bivalency introduces an avidity effect to the single-chain variable fragment (scFv)-AP fusion, making it resemble monoclonal IgG antibodies with two binding sites [17]. There are few published examples of mycotoxin assays utilizing AP as a fusion partner with a recombinant antibody fragment. Shu et al. (2016) [18] developed a competitive anti-idiotypic nanobody-AP fusion protein for the detection of Fumonisin B1 in cereals, and Liu et al. (2015) developed a competitive nanobody-AP fusion protein for setting up an Ochratoxin A assay [19]. ELISA methods utilizing scFv fused with AP have been reported for other low-molecular weight compounds besides mycotoxins, such as chloramphenicol [20], ractopamine [21] and clenbuterol [22]. Oyama et al. (2013) developed an assay for 11-Deoxycortisol, utilizing anti-idiotype scFv-AP antibodies that bind to the primary antibody variable binding site, competing with the analyte [23]. The target analyte blocks the binding of the anti-idiotype antibody, and so the scFv-AP antibody indicates indirectly the amount of the analyte in a sample. 

Competitive assay has been the most used assay format for mycotoxins and other small molecules, since two antibodies cannot bind them simultaneously due to their small size. Nevertheless, noncompetitive sandwich-type assays are known to have better sensitivity, kinetics and linear range compared to indirect competitive assays [24]. In order to circumvent the problem with small molecules, binding molecules able to recognize the IC formed by the primary antibody and the analyte have been developed, first through immunization [25], but later utilizing recombinant antibody [26] or peptide library techniques [27] and phage display. Recently, Omi et al. developed an ex vivo antibody development system called the Autonomously Diversifying Library (ADLib) system for generating chicken monoclonal antibodies against hapten-antibody IC [28].

**Figure 1:**
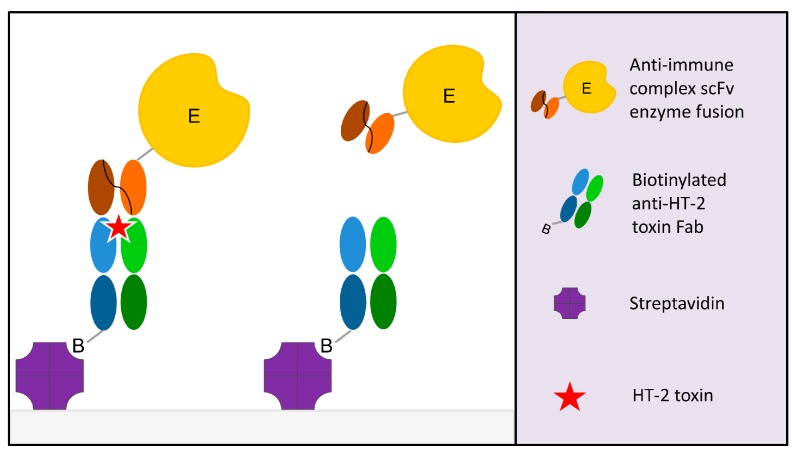
Principle of the noncompetitive immune complex ELISA assay. The anti-immune complex single-chain variable fragment alkaline phosphatase fusion (scFv-AP) binds to the immobilized anti-HT-2 Fab if HT-2 toxin is present in the sample. The positive readout is directly proportional to the HT-2 toxin concentration.

Previously, we reported an ultrafast noncompetitive fluorescence resonance energy transfer (FRET) immunoassay for HT-2, based on anti-IC Fab fragments [29]. In this article, we describe the first noncompetitive IC ELISA (Figure 1) that is specific for HT-2 only. Briefly, biotinylated HT-2 specific antibody Fab fragment is immobilized on a streptavidin-coated micro well. The sample is introduced together with scFv antibody-AP fusion that is specific for the complex, formed by the primary antibody and HT-2. The fusion enzyme is used for enabling a simple colorimetric noncompetitive assay for HT-2. The developed assay can detect HT-2 in a relevant concentration range from three different important cereal grain matrices, namely wheat, oats and barley.

## 2. Results and Discussion

### 2.1. Noncompetitive ELISA and Method Optimization

Anti-IC HT2-10 (H5) ScFv-AP fusion antibody (anti-HT-2-IC scFv-AP) was produced in *E. coli* bacteria and purified by metal affinity chromatography with a yield of 10.5 mg, purified scFv-AP fusion/L of culture medium without any optimization of the production protocol. The production yield was sufficient for noncompetitive ELISA assay development. The purity of the scFv-AP product was analyzed by sodium dodecyl sulfate polyacrylamide gel electrophoresis (SDS-PAGE) and no degradation products were observed. The amount and the ratio of the primary anti-HT-2 Fab and corresponding anti-HT-2-IC scFv-AP have an effect on the sensitivity of the assay. The amounts and the ratio of the antibodies were optimized and 500 ng of biotinylated anti-HT-2 Fab and 1500 ng of anti-HT-2-IC scFv-AP were chosen for real sample analysis.

### 2.2. Sensitivity of the Simple Noncompetitive ELISA in Buffer 

**Figure 2:**
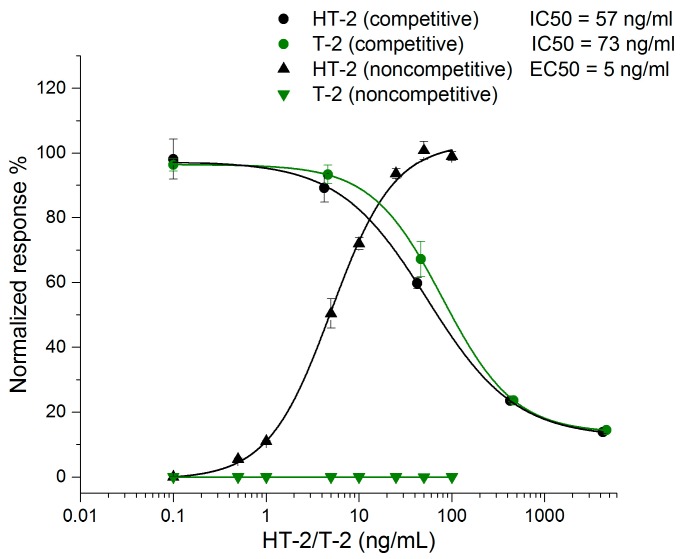
Comparison between the competitive ELISA assay and noncompetitive ELISA performed with spiked buffer (*n* = 3) (OriginPro 2016, logistic fit).

The sensitivity of the noncompetitive ELISA assay was compared to that of the corresponding competitive ELISA format in phosphate buffered saline (PBS, 15 mM sodium phosphate pH 7.3, 150 mM NaCl) (Figure 2). The half-maximal inhibitory concentration (IC50) value of the competitive ELISA method was 57 ng/mL for HT-2 and 73 ng/mL for T-2. The corresponding half-maximal effective concentration (EC50) value of the noncompetitive ELISA assay for HT-2 was 5 ng/mL, but for T-2 there was no observable response even with the highest concentration of 100 ng/mL, indicating the high specificity of the noncompetitive assay for HT-2 only. The noncompetitive ELISA was 10.8 times more sensitive for HT-2 compared to the competitive ELISA. The result is comparable with the sensitivity obtained earlier with the HT-2 FRET assay [29].

### 2.3. Effect of Methanol 

The effect of methanol (MeOH) as a solvent for the assay performance was studied with six concentrations of MeOH in water (2.5%, 5%, 7%, 10%, 14% and 20%) (Figure S1). 7% and 10% of MeOH showed the best linearity. With more than 10% of MeOH, the assay responses started to decline. 7% of MeOH, equivalent to a grain sample extracted with 70% MeOH-water and diluted 1:10 (*v*/*v*) with water, was chosen for further studies because of the good linearity and sensitivity in the HT-2 concentration range of 1–10 ng/mL. This concentration range is equivalent to 50–500 µg/kg of HT-2 in naturally contaminated grain samples.

### 2.4. Sample Incubation Time 

The sample incubation time affects the final assay response. The samples (0 ng/mL, 1 ng/mL and 5 ng/mL of HT-2 in 7% MeOH-water) were applied to the microtiter plate wells and incubated for 5, 15, 30 or 60 min with the anti-HT-2 Fab immobilized on the well. At low (1 ng/mL) HT-2 concentrations, no significant differences in responses from the abovementioned incubation times were observed. At a concentration level of 5 ng/mL, 60 min incubation yielded about 35% higher A405 response compared to 5 min incubation (Figure S2). In order to maintain sufficient sensitivity, 15 min incubation time was chosen for monitoring noncompetitive ELISA experiments for wheat and barley. For oats, 60 min sample incubation was selected because of the greater signal reduction effect of the oat matrix. 

### 2.5. Comparison of Alkaline Phosphatase Substrates

Two substrates, pNPP (Sigma, St. Louis, MO, USA) and BluePhos (KPL), were compared. After 10–15 min incubation, the A405 responses for both substrates were comparable. The advantage of BluePhos substrate was observed especially in longer incubation times. After 30 min, BluePhos gave 30% higher absorbance compared to pNPP for 10 ng/mL of HT-2 in 7% MeOH-PBS (Figure S3). BluePhos was selected to be used with real samples with 60 min color development in order to compensate for the signal reduction due to matrix effects of grain extractions.

### 2.6. Simple Noncompetitive Immune Complex ELISA with Real Samples 

**Figure 3:**
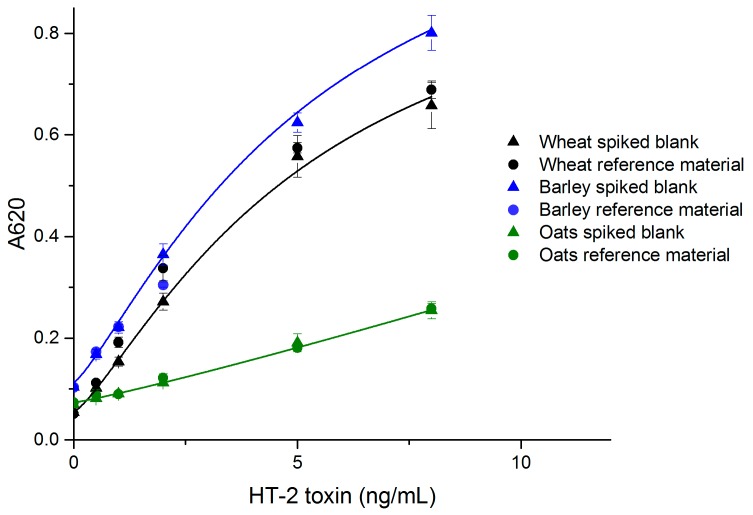
The correlation of naturally contaminated wheat, barley and oat sample dilutions with spiked blank standard curves (*n* = 6) (OriginPro 2016, logistic fit).

The performance of the developed simple noncompetitive IC ELISA with real samples was evaluated with wheat, barley and oats, which are known to be susceptible to type A trichothecene contamination. The standard curves made of spiked blank samples are presented in Figure 3. The limit of detection (LOD) values for wheat, barley and oats were 0.3 ng/mL (equivalent to 13 µg/kg grain), 0.1 ng/mL (4 µg/kg) and 0.3 ng/mL (16 µg/kg), respectively. The linear range for all grains was 25–400 µg/kg.

The recoveries and coefficients of variation (CV %) for wheat and oats were acceptable in the studied range (25–250 µg/kg for wheat and oats, and 25–100 µg/kg for barley) (Table 1). For barley, the available reference material had an HT-2 contamination of 111 µg/kg, and therefore the maximum reference sample concentration was 100 µg/kg. The sensitivity of the simple ELISA wheat assay was comparable to, or slightly better than that of the noncompetitive FRET-assay (LOD = 0.38 ng/mL) for wheat, which we reported previously [29]. Other reported HT-2/T-2 assays also have LODs in the same range as reported here. ELISA, immunomagnetic electrochemical array (ELIME) and SPR screening assays have been reported to achieve LODs of <10, 12, and 25 μg/kg, respectively [30]. Porricelli et al. [8] validated a fluorescence polarization assay for T-2 and HT-2 with LODs of 70 µg/kg (oats), 40 µg/kg (oat flakes and barley), 25 µg/kg (pasta) and 20 µg/kg (rye and oats crispbread). The simple noncompetitive ELISA method reported here is highly specific for HT-2 only. The reference samples for wheat and barley had almost four-fold higher T-2 concentration compared to HT-2 (Table 1). The satisfactory recoveries for HT-2 show that T-2 does not disturb the assay’s performance.

### 2.7. Matrix Effects 

Matrix effects are one of the greatest challenges in rapid diagnostics for small analytes. The overall response of the assay was lower with real samples, compared to the assay with a spiked buffer. Oat matrix decreased the signal more than wheat and barley matrices (Figure 3). The high fat content in oats might be responsible for the greater signal reduction effect in oat samples. The signal reduction effect was compensated by the more sensitive chromogenic substrate, and in the case of oat samples by optimized incubation time. It is also preferred to use a matrix-matched calibration curve for each grain in the case of quantitative measurements. The matrix effects for oats were compensated to some extent by using a 1 h sample incubation. The ELISA response could possibly be further enhanced by optimized sample preparation. 

## 3. Conclusions

To the best of our knowledge, we have developed the first simple noncompetitive IC ELISA test for HT-2 without cross-reactivity with the structurally very closely related *Fusarium* mycotoxin T-2. The assay was demonstrated to be functional in a relevant concentration range for all three tested grain matrices with matrix-matched calibration curves. By using a genetically fused detection enzyme (i.e., AP) and recombinant antibody fragment, the ELISA protocol is simplified and the hands-on time for the user is minimized. The primary antibody could be pre-coated on a microtiter plate, so the user only needs to add the extracted and diluted sample with the detection antibody, wash the plate after 15–60 min of incubation and add the substrate to develop a visible color that can be measured quantitatively with a spectrometer.

The sample preparation method was chosen on the basis of existing procedures for grain sample preparations for trichothecene mycotoxin analysis [11,31]. Assay parameters were optimized to obtain a fast assay with high sensitivity for real sample matrices. Matrix-matched calibration curves improved the accuracy of the simple ELISA test. By applying specialized sample preparation, the sensitivity could be further improved and the overall assay time reduced. The current EU recommendations are for the sum of HT-2 and T-2 [5]. Lattanzio et al. 2009 developed a method in which T-2 is hydrolyzed to HT-2 enzymatically before the measurement [32]. By using this method, the sum of both toxins could be measured using the developed HT-2 specific assay.

The simple ELISA method principle is applicable to other mycotoxins and small molecules that require specific detection. A similar assay utilizing specific anti-IC antibodies could also be developed, e.g., for other important trichothecenes (including T-2, deoxynivalenol and nivalenol), allowing an accurate multiplexed measurement of all toxins separately. The developed antibodies can also be applied to different biosensor platforms and antibody arrays and their properties, such as immobilization efficiency, can be further enhanced by antibody engineering.

## 4. Materials and Methods 

### 4.1. Chemicals, Reagents and Instrumentation

HT-2 and T-2 were purchased from Sigma Aldrich, as well as pNPP substrate. BluePhos^®^ Microwell Phosphatase Substrate System was obtained from KPL, Gaitersburg, MD, USA. Diethanolamine-MgCl_2_ buffer for AP was purchased from Reagena (Toivala, Finland). Naturally contaminated reference wheat (455 ± 89 µg HT-2/kg and 1769 ± 355 µg T-2/kg) and barley (111 ± 22 µg/kg HT-2 and 442 ± 90 µg/kg T-2) samples were purchased from Aokin (Berlin, Germany). Naturally contaminated oat sample (1826 µg/kg HT-2; 548 µg/kg T-2 and 300 µg/kg HT-2-3-glucoside) and blank samples for all three grains were provided by the Finnish Food Safety Authority (EVIRA; Helsinki, Finland). The blank samples were analyzed by liquid chromatography tandem mass spectrometry (LC-MS/MS) for absence of HT-2, T-2 or HT-2-3-glucoside [33]. Kaivogen Kaisa96 streptavidin plates were purchased from Kaivogen (Turku, Finland). Escherichia coli XL-1 blue cells were purchased from Agilent Technologies Inc. (Cedar Creek, TX, USA). Superb broth (SB, 2% yeast extract, 3% tryptone, 1% MOPS, pH 7) and Luria Broth (LB; 0.5% yeast extract, 1% tryptone, 1% NaCl) supplemented with 50 µg/mL kanamycin were used as cultivation medium and for preparing agar plates, respectively. Terrific Broth (TB; 1.2% yeast extract, 2.4% soy peptone, 1.25% K_2_HPO_4_·3H_2_O, 0.23% KH_2_PO_4_, 0.4% glycerol) was used as production medium for scFv-AP fusion proteins. A SPECTROstar Omega spectrometer (BMG Labtech, Ortenberg, Germany) was used for reading the ELISA plates.

### 4.2. Primary and Secondary Antibody Development

The development of primary and secondary antibody is described in [29]. Briefly, the primary antibody anti-HT-2 Fab was selected using phage display from an immunized library. HT-2-blue protein conjugate was used as an immunogen. The HT-2 specific antibodies were selected against HT-2-AP conjugate using magnetic beads and screened utilizing a robotic station and HT-2-human serum albumin (HSA) conjugate as the antigen. The primary antibody anti-HT-2 Fab was characterized using a Biacore Q biosensor to determine the IC50 value and cross-reactivity with closely related mycotoxins such as T-2, T-2-triol, T-2 tetraol, diacetoxyscirpenol, 15-acetyldeoxynivalenol, 3-acetyldeoxynivalenol, deoxynivalenol, deoxynivalenol-3-glucoside, nivalenol and neosolaniol. Secondary anti-IC antibody was selected from a naïve scFv phage display library against a complex formed by anti-HT-2 Fab and HT-2 attached in an oriented manner through a six-histidine tag to magnetic beads. The screening of the positive anti-IC antibodies was performed by phage ELISA. 

### 4.3. Construction of The ScFv-AP Plasmid

**Figure 4:**
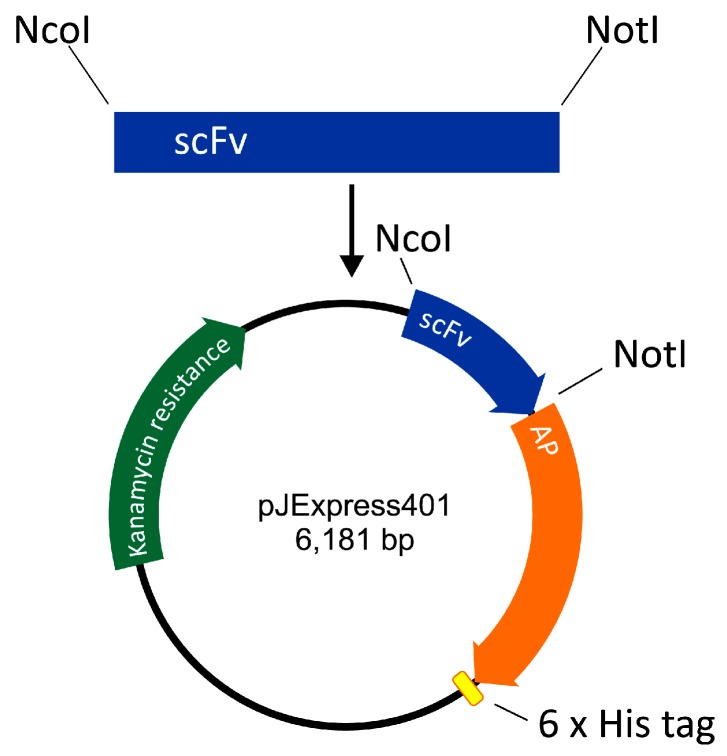
Construction of single-chain variable fragment alkaline phosphatase fusion (scFv-AP).

The scFv-AP-His6 construct was designed as follows. DNA of pelB signal sequence was designed to include a restriction site for NcoI. The amino acid sequence of *E. coli* AP was retrieved from UniProt (PPB_ECOLI) and corresponding DNA was codon-optimized by DNA 2.0 (CA). A DNA restriction site for NotI was inserted between scFv and AP sequences, and His6 sequence with a stop codon was inserted at the 3′ end of the AP. The construct thus formed was ordered from DNA 2.0 in the expression vector pJExpress401 harboring gene for kanamycin resistance and a T5 promoter for expression of the anti-HT-2 IC scFv-AP fusion protein (Figure 4).

### 4.4. Cloning of ScFv-AP Fusion Proteins

Anti-HT-2-IC scFv was cloned from an antibody library vector [11] to pJExpress401 including AP protein. The scFv insert was digested using NcoI and NotI restriction enzymes and ligated to twice digested pJExpress401 (Figure 4). Successful cloning was confirmed by sequencing of the nucleotide sequence of the fusion protein (GATC Biotech, Konstanz, Germany).

### 4.5. Production and Purification of Anti-HT-2 IC ScFv-AP Fusion Proteins

The anti-HT-2 IC scFv-AP in XL1-Blue *E. coli* strain was inoculated to 50 mL of terrific broth (TB) medium (50 µg/mL kanamycin, 10 µg/mL tetracyclin and 1% glucose) and incubated overnight in +37 °C with shaking at 250 rpm. The cultivation was diluted 1:50 (*v*/*v*) in 330 mL of TB (50 µg/mL kanamycin, 10 µg/mL tetracyclin and 0.1% glucose) and cultivated in +37 °C and 220 rpm for 5 h. The production of scFv-AP was induced with 1 mM IPTG and at the same time the concentration of antibiotics was doubled. Cultivation was continued at 30 °C overnight with shaking at 230 rpm. The cells were harvested by centrifugation at 8000 rpm for 20 min at 4 °C. 2 mg DNase I/L of supernatant was added for removing unwanted DNA from lysed cells, improving the protein extraction efficiency. After incubation for 1 h at 37 °C, the supernatant was separated by centrifugation at 8000 rpm for 20 min at 4 °C and filtered through Whatman GF/C filter paper. The soluble scFv-AP was purified from the supernatant by metal affinity chromatography according to the manufacturer’s instructions (GE Healthcare, Chicago, IL, USA) and the purity was analyzed by SDS-PAGE.

### 4.6. Competitive HT-2 ELISA

The sensitivity of the competitive ELISA for HT-2 and T-2 was evaluated as follows. Maxisorp 96-well plates (Nunc) were coated with 10 ng of HT-2-HSA conjugate in 100 µL 0.1 M Na-bicarbonate buffer, pH 9.6 per well overnight at 4 °C [29]. Anti-HT-2 Fab (0.25 µg/mL) was pre-incubated with 10 pM-10 µM concentrations of HT-2 or T-2 for 1 h (2% DMSO, Superblock (Thermo Scientific, Waltham, MA, USA) with 0.05% Tween20). 100 µL of the samples were applied on an ELISA plate and incubated for 1 h. The plate was washed three times with PBST (PBS, 0.05% Tween 20). 100 µL of pNNP (2 mg/mL) in Diethanolamine-MgCl_2_ buffer was added and the absorbance at 405 nm was measured after 60 min. The inhibition curve was fit and the IC50 value was calculated using logistic fit in OriginPro 2016 software (b9.3.226, Northampton, MA, USA, 2016).

### 4.7. Noncompetitive Immune Complex ELISA and Method Optimization

A streptavidin plate (Kaisa96, Kaivogen) was coated with biotinylated anti-HT-2 Fab antibody for 30 min at room temperature. After washing three times with 300 µL of PBST, the sample with anti-HT-2-IC scFv-AP was added and incubated for 1 h at room temperature. After washing three times with 300 µL of PBST, 100 µL of BluePhos substrate was added according to the manufacturer’s instructions and absorbance at 620 nm was measured with an Omega spectrometer. The ratio of the immobilized anti-HT-2 Fab and anti-HT-2-IC scFv-AP was optimized by studying the signal/background ratio of 7 ng/mL HT-2 in PBS buffer versus plain buffer. Four coating concentrations (500 ng/well; 300 ng/well; 100 ng/well and 50 ng/well) were assayed with four concentrations of anti-HT-2-IC scFv-AP (700 ng/mL; 500 ng/mL; 300 ng/mL and 100 ng/mL). The sample incubation time was optimized by performing the assay for 5, 15, 30 and 60 min in buffer (PBS) and comparing the sensitivities of two chromogenic substrates, pNPP (Sigma) and BluePhos (KLP), for AP according to the manufacturer’s instructions. The effect of MeOH on the assay performance was studied by applying three concentrations of HT-2 (0, 1, 5 and 10 ng/mL) with differing MeOH concentrations in water (0%; 2.5%; 5%; 7%; 10%; 14% and 20%). Matrix effects were studied by comparing the assay with 7% MeOH-water with 1:10 (*v*/*v*) diluted wheat sample (final MeOH concentration 7%). The performance of the simple ELISA assay with real samples was evaluated using three grain matrices: wheat, barley and oats. Five samples (0, 25, 50, 100 and 250 µg/kg) for wheat and oats and four samples for barley (0, 25, 50 and 100 µg/kg) were prepared by weighing and mixing the reference sample with the blank sample. Standard curves were generated by spiking blank grain extracts with equivalent HT-2 concentrations (0, 0.5, 1, 5 and 8 ng/mL). The LOD was calculated as: LOD = blank average + [3 × blank standard deviation]. The EC50 value was calculated using OriginPro 2016 software and logistic fit.

### 4.8. Sample Preparation

Sample preparation was performed according to Arola et al. 2016 [29]. Briefly, 5 g of each ground grain sample was weighed and extracted with 25 mL of 70% MeOH-water. After the extraction for 1 h at room temperature with rotation, the samples were centrifuged for 10 min at 6000 *g* and filtered through Whatman GF/C filter paper. For analysis, the samples were diluted 1:5 in PBS (15 mM sodium phosphate pH 7.3, 150 mM NaCl). The final dilution of the sample was 1:10 (*v*/*v*) when mixed 1:1 (*v*/*v*) with anti-HT-2-IC scFv-AP antibody dilution.

## Figures and Tables

**Table 1:** Noncompetitive HT-2 toxin ELISA performance with cereal samples.

Cereal Grain	Reference Samples		Noncompetitive ELISA Performance		
	T-2 ± SR ^1^ (µg/kg)	HT-2 ± SR ^1^ (µg/kg)	HT-2 ± SD (µg/kg) *n* = 6	Recovery (%)	CV (%) ^3^
**Wheat**	98 ± 20	25 ± 5	30 ± 1	122	4
	195 ± 39	50 ± 10	64 ± 4	129	9
	390 ± 78	100 ± 20	131 ± 13	131	13
**Barley**	100 ± 20	25 ± 5	28 ± 2	112	8
	199 ± 40	50 ± 10	46 ± 2	92	4
	398 ± 80	100 ± 20	78 ± 2	78	2
**Oats ^2^**	8	25	33 ± 4	133	16
	15	50	45 ± 8	90	16
	30	100	119 ± 18	119	18
	75	250	253 ± 7	101	3

^1^ SR = Satisfactory range for reference samples from Aokin; ^2^ Oat reference sample analyzed with liquid chromatography tandem mass spectrometry (LC-MS/MS), satisfactory range not determined; ^3^ CV (%) = Coefficient of variation.

## Supplementary Materials

The following are available online at www.mdpi.com/2072-6651/9/4/145/s1, Figure S1: Noncompetitive HT-2 toxin ELISA: Effect of MeOH to the assay performance. Figure S2: Noncompetitive HT-2 toxin ELISA: Effect of sample incubation time on the assay response with spiked 7% MeOH-water. Figure S3: Noncompetitive HT-2 toxin ELISA: Comparison of two colorimetric substrates for alkaline phosphatase in 7% MeOH-PBS.
