# Supplementary material for: A Simple and Specific Noncompetitive ELISA Method for HT-2 Toxin Detection
Source: Toxins (Basel). 2017 Apr 20;9(4):145. doi: 10.3390/toxins9040145 (PMC5408219; doi:10.3390/toxins9040145)
Supplement: Supplementary file 1 [file toxins-09-00145-s001.pdf]

Supplementary Materials: A Simple and Specific Noncompetitive ELISA Method for HT-2 toxin Detection

Henri O. Arola, Antti Tullila, Alexis V. Nathanail, Tarja K. Nevanen

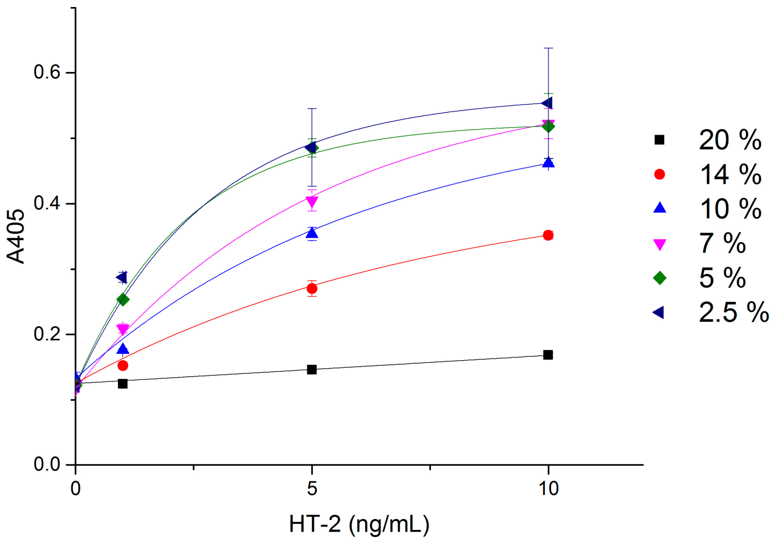

Figure S1. Noncompetitive HT-2 toxin ELISA: Effect of MeOH to the assay performance.

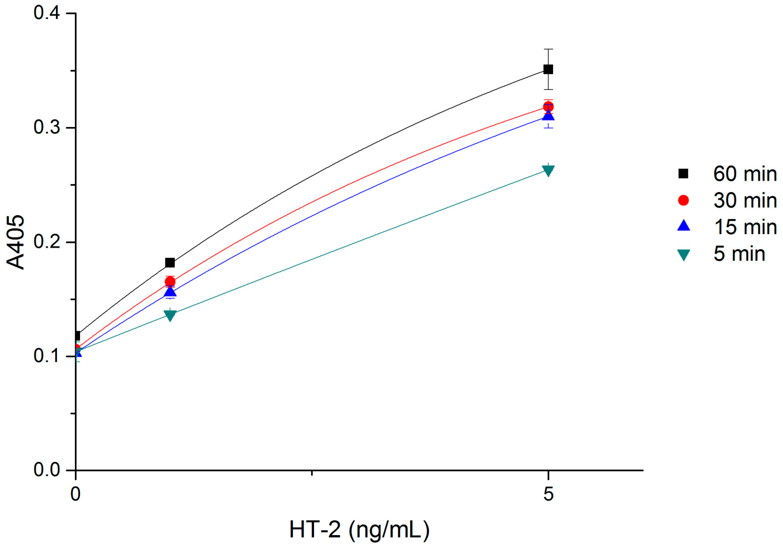

Figure S2. Noncompetitive HT-2 toxin ELISA: Effect of sample incubation time on the assay response with spiked 7% MeOH-water.

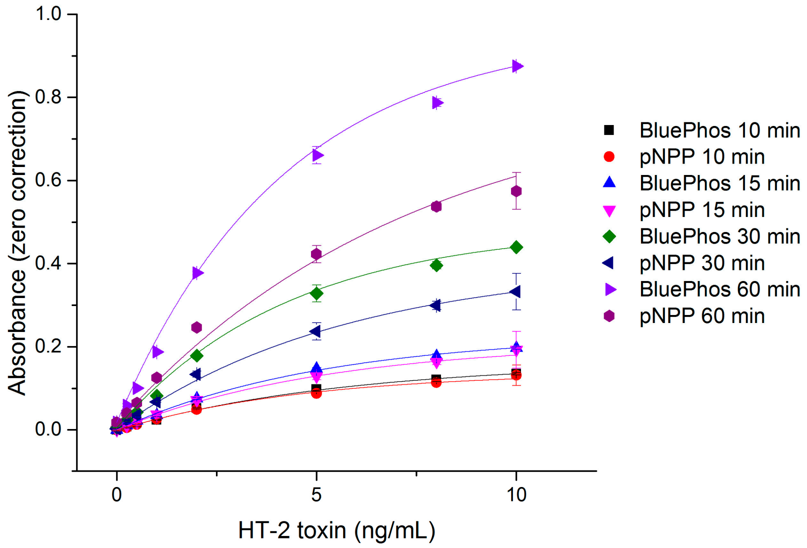

Figure S3. Noncompetitive HT-2 toxin ELISA: Comparison of two colorimetric substrates for alkaline phosphatase in 7% MeOH-PBS.
